# Risk Factors and Pharmacological Interventions Impacting Cerebrovascular Ischemic Events in Giant Cell Arteritis: A Narrative Review

**DOI:** 10.1002/iid3.70122

**Published:** 2025-01-16

**Authors:** Muhammad Osama Siddiqui, Mohammad Ali Syed, Ayaan Ahmed Qureshi, Mustafa Hussain Imam, Jatin Motwani, Verkha Kumari, Arooba Siddiqui, Noor Ul Ain, Mohammed Hammad Jaber

**Affiliations:** ^1^ Liaquat National Hospital and Medical College, MBBS Karachi Pakistan; ^2^ Karachi Medical and Dental College, MBBS Karachi Pakistan; ^3^ Faculty of Medicine Alzaiem Alazhari University Khartoum Sudan

## Abstract

**Introduction:**

Giant cell arteritis (GCA) is a common vasculitis predominantly affecting larger vessels, especially in individuals aged 70–79. Cerebrovascular ischemic events (CIE), such as stroke and transient ischemic attacks, are serious but rare complications of GCA, with a pooled prevalence of 4%. Some studies found that within 2 weeks of GCA diagnosis, 74% and 34% of patients experience transient or severe ischemic events, respectively.

**Aims:**

Our study aims to help physicians better manage GCA patients to reduce GCA‐related CIE by indicating important risk factors and pharmacological intervention to prevent GCA‐related CIE, particularly in the first few days of diagnosis when the risk of CIE is highest.

**Methods:**

A comprehensive literature search was conducted using Pubmed, Google Scholar, Scopus, and other relevant medical databases. As this study was a narrative review, the literature search was done in a nonsystematic manner. Studies published from 2000 to 2024 were reviewed in a nonsystematic manner for information on incidence, pathology, risk factors, pharmacological intervention, and management of GCA‐related CIE.

**Results:**

Findings indicate that age, male gender, hypertension, and smoking significantly increase the risk of GCA‐related CIE, while factors such as anemia, higher body mass index (BMI), and elevated inflammatory markers (C‐reactive protein [CRP] and erythrocyte sedimentation rate [ESR]) appear to have protective effects. Symptoms of ischemia in the ophthalmic artery were identified as the strongest predictors of CIE. Pharmacological treatments, including glucocorticoids and tocilizumab, are instrumental in managing and potentially preventing CIE in GCA patients, with adjunctive therapies such as aspirin and antiplatelet agents also showing promise.

**Conclusion:**

GCA‐related CIE such as stroke can be very debilitating and deadly conditions, particularly when GCA is initially diagnosed. However, with early diagnosis and proper management of risk factors, GCA‐related CIE can be prevented and its severity can be reduced. Ischemia in the ophthalmic artery is found to strongly predict GCA‐related CIE while aspirin and antiplatelet agent during the first 3 months may prevent GCA‐related CIE. Risk factors such as BMI and smoking may help in stratifying the risk of GCA‐related CIE. This review underscores the importance of further studies with detailed, well‐designed approaches to risk factor analysis to strengthen these associations. Identifying these risk factors is crucial for reducing morbidity and mortality, equipping physicians to better assess and mitigate the risk of CIE in GCA patients.

## 1 Introduction

A granulomatous vasculitis known as Giant Cell Arteritis (GCA) typically affects medium‐ to large‐sized vessels, particularly those branching from the aorta, with the superficial temporal artery often involved [1]. It has a peak incidence ranging from the age of 70–79; it is the most common kind of systemic necrotizing vasculitis, with higher chances of developing it with increasing age [2]. There are 10.00 cases/100,000 of GCA reported as the pooled incidence and 51.74 cases/100,000 recorded as the pooled prevalence among individuals over 50 [3].

The gold standard for diagnosing a patient with GCA is a temporal artery biopsy. However, the American College of Rheumatology (ACR) has developed a set of criteria commonly used for clinical diagnosis. The ACR criteria [4], originally developed in 1990, were enhanced for improved sensitivity in the 2022 American College of Rheumatology/European League Against Rheumatism (ACR/EULAR) criteria [4]. The ACR/EULAR criteria are typically applied once a medium or large vessel vasculitis is diagnosed and similar disorders are excluded. It's important to note that while the ACR criteria are essential for clinical research, they are not mandatory for clinical diagnosis, despite their widespread use as a diagnostic tool. The 2022 ACR/EULAR criteria are outlined below [4] (see Appendix A).

Transient ischemic attack (TIA) and stroke are examples of cerebrovascular events (CVE), which are major clinical consequences of GCA [5]. According to a meta‐analysis carried out in 2023 by Thomas Penet and associates, the combined occurrence of cerebrovascular ischemic events (CIE) linked to GCA was found to be 4% [6]. In addition, a 2016 meta‐analysis by Patompong Ungprasert and colleagues revealed that, in comparison to non‐GCA comparators, patients with GCA had a pooled risk ratio for CVE of 1.40 (95% CI 1.27–1.56) [7].

Moreover, a 2015 study by Samson et al. found that the probability of cerebrovascular ischemia episodes in GCA patients was 0.76 per 100,000 patient‐years [8]. Consequently, the reported prevalence of cranial involvement in GCA ranges from 1.5% to 12.95% [6]. These findings underscore the significance of understanding the incidence and prevalence of cerebrovascular complications in the context of GCA [6]. Identifying risk factors for CIE in GCA is crucial, given their huge impact on morbidity and mortality rates [6]. The risk factors for CIE in patients diagnosed with GCA can be attributed to traditional vascular pathologic reasons or to the pathognomonic reasons of GCA itself. This narrative review delves into the spectrum of associated risk factors alongside pharmacological interventions.

### 1.1 Stroke As a Common Manifestation of Cerebrovascular Disease (CIE)

Stroke is an uncommon but potentially dangerous complication of GCA, with low levels of acute phase reactants, coronary heart disease, male gender, and concurrent symptoms of ischemic ophthalmia being the main risk factors for stroke in individuals with GCA [9]. Most GCA‐associated mortality is due to strokes, and most deaths occur 3 months or less after the stroke [10]. Unlike strokes brought on by atherosclerosis, strokes related to GCA usually impact the vertebrobasilar arterial system [9].

A retrospective analysis was performed on 287 patients whose GCA was verified by biopsy. The objective of the analysis was to ascertain the prevalence and risk variables for vertebrobasilar strokes at the time of diagnosis. Eight individuals (2.8%) experienced strokes, of which 87.5% had vertebrobasilar strokes, according to the study. Males were shown to be more likely to suffer from vertebrobasilar strokes, as evidenced by the fact that six out of seven patients had this condition. Individuals with vertebrobasilar strokes had a higher risk of permanent blindness when compared with other GCA patients (42.9% vs. 11.8%, *p* = 0.05). This elevated risk is likely due to the inclusion of arteries supplying the eye stemming from the internal carotid artery. Permanent vision loss (OR, 5.42; 95% CI, 1.26–23.39) and arterial hypertension (OR, 5.06; 95% CI, 1.02–25.12) were the greatest indicators of stroke. Stroke risk was considerably lower in women (OR, 0.10; 95% CI, 0.04–0.26). The study also found that corticosteroid medication was started primarily after strokes that included the vertebrobasilar area. This study demands the need to treat traditional cardiovascular risk factors in this population by offering insightful information on the prevalence and risk variables linked to strokes in GCA patients [9].

In an effort to speed up the diagnosis of GCA, Simon Parreau's study sought to differentiate the features of GCA‐related strokes from those of GCA cases without strokes and strokes brought on by atherosclerosis or embolism. Nineteen members of a cohort that had their strokes connected to GCA and were followed up from 1982 to 2021 were included in the study. Forty patients over 50 who had typical first‐ever strokes and 541 GCA patients who did not have a stroke made up the control groups. A review of clinical, laboratory, and imaging outcomes related to GCA‐associated strokes revealed that while patients with these strokes had fewer headaches and scalp discomfort, they also had higher rates of comorbidities and aortitis on imaging. Anomalies in external carotid ultrasonography (ECU), high C‐reactive protein (CRP) values greater than 3 mg/dL, and the lack of anterior circulation involvement were all found to be independently associated with GCA‐related strokes. The early survival rate for stroke patients with GCA‐related strokes dropped significantly when compared with control GCA patients with and without strokes. Evaluating these characteristics may improve the prognosis for GCA‐related strokes and decrease diagnostic delays by promptly informing clinicians of the stroke site, ECU results, and CRP levels [10].

A retrospective multicenter cohort analysis involving 200 healthy controls and 40 stroke patients with GCA was carried out to clarify the goal of the current investigation. By comparing these groups, the study aimed to gain additional insights into the characteristics and incidence of strokes associated with GCA. Among the subjects, there were 21 women (53%) with a median age of 78 (60–91) years who had experienced strokes associated with GCA. According to other studies, the cohort had a prevalence of GCA‐related strokes ranging from 3% to 7%. When the GCA was diagnosed, 73% of the stroke patients had already experienced a stroke, with the vertebrobasilar region being impacted in 73% of those cases. Seven people died shortly after their strokes started. In those suffering from stroke, ocular ischemia symptoms were reported more frequently [25 (63%) vs. 50 (25%), *p* < 0.001]. The greatest predictor of the likelihood of a stroke, according to multivariate logistic regression, was the existence of ocular ischemic symptoms at diagnosis (OR 5, 95% CI 2.14–12.33, *p* = 0.0002). Seventy‐three percent of cases with neurovascular imaging showed involvement of the vertebrobasilar artery, while three patients showed involvement of the anterior cerebral artery region. The three risk variables that were most frequently seen in individuals with GCA‐related strokes were smoking, hypertension, and high cholesterol. These findings emphasize the necessity of detecting ocular symptoms and monitoring anemia in GCA patients for the purpose of minimizing the risk of stroke. To investigate the frequency and features of arterial involvement in GCA‐related stroke, more research is required [11].

In another study, acute cerebral ischemia episodes were observed in 18 (16%) of the 129 GCA patients who were identified between September 2010 and October 2018, according to a multicentric retrospective review. Compared with patients without strokes, stroke patients appeared to have been older (median age 83 vs. 76 years; *p* = 0.014) and increasingly often male (61% vs. 30%; *p* = 0.014). In stroke patients, pathology involving the anterior optic nerve was associated with a greater frequency (33% vs. 11%; *p* = 0.02). In seven cases, ischemic episodes happened right after the GCA diagnosis, and in 11 cases, they happened a year later. More than 80% of patients experienced GCA symptoms following diagnosis, including acute anterior neuropathy (6%), polymyalgia rheumatica symptoms (2%), and headaches (89%). High concentrations of acute‐phase reactants were also found at the stroke site in 83% of cases. Male sex, advanced age, a history of diabetes, and concurrent anterior ischemic optic neuropathy were risk factors for GCA patients. An estimated 40%–60% of GCA strokes were thought to be caused by involvement of the vertebrobasilar artery, with ischemic lesions primarily found in the vertebrobasilar (61%) and carotid (28%) areas. The internal carotid artery (1 patient), the basilar artery (2 cases), the circle of Willis (2 cases), and the vertebral arteries (11 patients) all had stenosis and/or occlusions found by vascular imaging. These results highlight the need for more investigation into the treatment of strokes caused by GCA, given their substantial effects on life expectancy as well as quality of life [12].

A fifth of GCA patients in a large prospective study with 295 new cases had severe cranial ischemic consequences (sCIC), which included stroke and eye problems. Because of the related morbidity and mortality, sCIC continues to be a major problem, despite greater illness awareness and faster diagnosis. Finding sCIC risk factors in GCA was the goal to enhance the long‐term prognosis. Growing older was found to be substantially correlated with sCIC (OR 1.08, 95% CI: 1.04, 1.13, *p* < 0.001), according to the investigations. The most significant predictor of sCIC was determined to be jaw claudication (OR 3.43, 95% CI: 1.84, 6.42, *p* < 0.001), emphasizing the significance of this hallmark symptom of GCA. Moreover, smoking was found to be a significant risk factor (OR 1.92, 95% CI: 1.01, 3.65, *p* = 0.046); on the other hand, a slightly lower risk was associated with a greater quantity of CRP (OR 0.99, 95% CI: 0.99, 1.00, *p* = 0.011). Clinically, ESR (OR 0.99, 95% CI: 0.99, 1.00, *p* = 0.015) and CRP levels (OR 0.99, 95% CI: 0.99, 1.00, *p* = 0.016) indicated lower inflammatory activity in patients with sCIC than in those without sCIC. Hemoglobin concentrations were also greater in the sCIC group (OR 1.00, 95% CI: 1.00, 1.01, *p* = 0.014). The study did not find any evidence of a substantial correlation between extracranial large artery involvement and severe ischemic vision problems in relation to arteries implicated in sCIC. Nevertheless, patients with obvious neurological abnormalities were the only ones eligible for comprehensive neurological workups and neuroimaging, which may have caused the prevalence of stroke to be underestimated. Among the few people in the cohort with GCA‐related strokes, the vertebrobasilar artery area (4/9, 44%) witnessed considerably more strokes than a stroke caused by atherosclerosis, which impacts the vertebrobasilar region in 15%–20% of cases. The study provides valuable information on the drivers of sCIC in GCA, underscoring the significance of close monitoring and tailored treatment approaches for patients who are at risk [13].

In a well‐phenotyped cohort, the study conducted at Lille University Hospital evaluated the traits and frequency of cerebrovascular ischemic episodes (CIE) associated with GCA. A meta‐analysis of past research was also added to provide compelling evidence of the connection between GCA and stroke. Out of the 339 people who underwent screening, 271 (33% of the total) had a mean age of 72.9 years and satisfied the ACR diagnostic criteria for GCA. The study found that the cohort had a 5.2% prevalence of GCA‐related CIE; however, the pooled frequency of 4% (95% CI 3%–6%) was revealed by the meta‐analysis. In spite of the fact that these differences were not statistically significant, patients with GCA‐related CIE had a lower body mass index (BMI) (22.5 vs. 24.9 kg/m^2^, *p* = 0.02) and were more likely to be male (57% vs. 32%, *p* = 0.075) and smokers (54% vs. 30%, *p* = 0.12). Other conventional vascular risk variables like dyslipidemia, arterial hypertension, and diabetes did not change appreciably.

Clinical manifestations of CIE patients associated with GCA included raised inflammatory markers such as CRP, polymyalgia rheumatica, headache (71%), jaw claudication, visual complaints, and constitutional symptoms. The study found several significant arterial involvements, including vertebral artery thrombosis detected by Doppler ultrasound (17% vs. 0.8%, *p* = 0.012), vertebral artery involvement (50% vs. 3.4%, *p* < 0.001), intracranial artery involvement (50% vs. 1.8%, *p* < 0.001) on computed tomography angiography (CTA) and/or magnetic resonance angiography (MRA), axillary artery involvement on positron emission tomography (PET)/computed tomography (CT) (55% vs. 20%, *p* = 0.016), ophthalmic artery involvement, subclavian artery involvement (42%), abdominal aorta involvement (54%), and thoracic aorta involvement (51%). These findings suggest that, in addition to clinical characteristics, lower BMI, male gender, smoking status, and specific vascular involvements may be risk factors for CIE associated with GCA. Further research is needed to validate these findings and examine the possible mechanisms behind these connections [6].

A pilot research examining the relationship between ischemic stroke and GCA that affects the vertebrobasilar region of the brain's arteries was conducted in 2021. Assessments of 65 patients with vertebrobasilar strokes were included in the study. According to the study, 3.1% of cases of GCA were reported. Patients with GCA had decreased hemoglobin levels (10.4 compared with 14.6 mg/dL, *p* = 0.003), increased erythrocyte sedimentation rate (ESR) (median, 75 vs. 11 mm in 1 h, *p* = 0.001), and higher CRP levels (3.84 *v*s. 0.25 mg/dL, *p* = 0.01). Additionally, in the vertebrobasilar area, they had a higher likelihood of multiple stenoses or occlusions (100% vs. 7.9%, *p* = 0.01). The study's analysis supported these findings, indicating that GCA patients also tended to be older, experienced headaches, had high markers of inflammation, anemia, and multiple vertebrobasilar stenoses or occlusions (of which 70% were afflicted). It was estimated that 2%–2.6% of patients with vertebrobasilar strokes had GCA. The study also found that among GCA patients, diabetes mellitus, dyslipidemia, hypertension, atrial fibrillation, and older age were common risk factors. Nevertheless, precise prevalence rates were not given [14] (Table 1).

**Table 1:** Overview of studies investigating giant cell arteritis (GCA)‐related cerebrovascular ischemic events (CIE).

References	Study design	Sample size	Prevalence of GCA‐related CIE	Risk factors for GCA‐related CIE	Clinical characteristics
Elhfnawy et al. [14]	Prospective Cohort	65 patients	3.10%	Older age, headaches, elevated inflammatory markers, anemia, multiple stenoses or occlusions in vertebrobasilar area	Higher ESR and CRP (*p* = 0.001, *p* = 0.01 respectively), low hemoglobin (*p* = 0.003), higher likelihood of multiple stenoses or occlusions in vertebrobasilar area (*p* = 0.01)
Parreau et al. [10]	Retrospective Cohort	560 patients	3.40%	Absence of anterior circulation involvement, abnormalities in external carotid ultrasound, CRP levels > 3 mg/dL	Majority of strokes in vertebrobasilar system, lower incidence of headache (*p* < 0.01), higher frequency of concomitant diseases (*p* = 0.03)
Hočevar et al. [13]	Prospective Cohort	295 patients	20% (severe cranial ischemic consequences)	Older age (OR 1.08, 95% CI: 1.04–1.13, *p* < 0.001), jaw claudication (OR 3.43, 95% CI: 1.84–6.42, *p* < 0.001), smoking (OR 1.92, 95% CI: 1.01–3.65, *p* = 0.046), higher CRP levels (OR 0.99, 95% CI: 0.99–1.00, *p* = 0.011), elevated hemoglobin concentrations (OR 1.00, 95% CI: 1.00–1.01, *p* = 0.014)	More strokes involving the vertebrobasilar artery area, less inflammatory activity (ESR: OR 0.99, 95% CI: 0.99–1.00, *p* = 0.015; CRP: OR 0.99, 95% CI: 0.99–1.00, *p* = 0.016), no significant correlation with extracranial big artery involvement
Pariente et al. [12]	Multicentric Retrospective Study	129 patients	16%	Older age, male sex, history of diabetes, concomitant anterior ischemic optic neuropathy	Majority of strokes in vertebrobasilar and carotid areas, arterial stenosis/occlusions found in various arteries (*p* < 0.05 for arterial stenosis/occlusions)
de Boysson et al. [11]	Retrospective Multicenter Case‐control Study	200 patients control, 40 patients with GCA related CIE	—	Lack of anemia, ocular ischemia symptoms at diagnosis	Majority of strokes affect vertebrobasilar area, reduced CRP levels (*p* = 0.04), less anemia (*p* = 0.03), more ocular ischemia symptoms (*p* < 0.001)
Gonzalez‐Gay et al. [9]	Retrospective review of the case records of all patients diagnosed with biopsy‐proven GCA	287 patients	2.80%	Low acute phase reactants, coronary heart disease, male gender, concurrent symptoms of ischemic ophthalmia	Vertebrobasilar strokes most common (87.5%), higher smoking history (six out of seven patients with vertebrobasilar strokes were male), irreversible visual loss as a result of arteritic involvement of ocular branches from internal carotid (42.9% vs. 11.8% in nonstroke patients), hypertension more prevalent in stroke patients (although not highly significant, *p* = 0.05), higher hemoglobin levels in patients with vertebrobasilar strokes (13.2 ± 1.5 g/dL vs. 11.7 ± 1.6 g/dL in those without strokes, *p* = 0.009)

## 2 Pathophysiology of GCA

Genetic variables such as HLA alleles and non‐HLA genetic loci cause a complex immune response that leads to GCA. Once dendritic cells are activated due to an unknown cause, they also activate CD4 + T cells. Interleukin‐6 (IL‐6), IL‐12, IL‐17, and IL‐18 are pro‐inflammatory cytokines that cause CD4+ T cells to develop into Th1 and Th17 cells, and these cells, along with macrophages, infiltrate the vascular adventitia and cause granulomatous inflammation. This further leads to intimal hyperplasia and luminal occlusion. The ultimate result of this process is an increased risk of aneurysm formation, occlusion, or stenosis in the impacted arteries, which may result in CVE such as stroke [15, 16, 17, 18].

### 2.1 Hypertension

A close relationship was found between hypertension and stroke in 57 patients diagnosed with GCA in retrospective research conducted in Dijon between 2001 and 2012. The reported incidence of GCA was 10.9/100,000/year for people over 50 and 13.4/100,000/year for people over 55. Out of 57 individuals with biopsy‐proven GCA, four (7%) had a stroke diagnosis. All stroke patients who had previously undergone a GCA had elevated blood pressure. There was a glaring disparity in gender, with men making up 75% of cases. The majority of strokes occurred in the vertebrobasilar area. A GCA‐related stroke was more common in men (1.36/100,000/year) than in women (0.33/100,000/year). These results highlight how crucial it is to comprehend how GCA interacts with stroke and hypertension to effectively manage these conditions [8].

In a study conducted by Gonzalez in 2009, individuals who had previously experienced hypertension before being diagnosed with GCA demonstrated a noticeable increase in the risk of stroke (odds ratio [OR] = 5.06; 95% confidence interval [CI] = 1.02–25.12; *p* = 0.05). This finding implies that people who had hypertension before being diagnosed with GCA are at a higher risk of having strokes after receiving a GCA diagnosis [9].

Managing blood pressure is essential for the endovascular treatment for the refractory GCA, a targeted systolic blood pressure of ≤ 120 mmHg postprocedure to optimize the treatment outcomes and minimize the risk of ischemic complication [19]. Noncompliance with medication is a major aspect to consider when treating patients with chronic conditions such as hypertension. Noncompliance with medication varies over different age groups and locations. In 2015, 24% of patients aged 65–74 years were noncompliant in America [20]. Another study found a greater prevalence of nonadherence to medication in patients over 80 years old [21]. Physicians must consider this while treating GCA patients, as 70–79 years is the peak age range for incidence.

### 2.2 BMI

The Prognosis, morbidity, and complications of most diseases have always been influenced by the patient's BMI. Either underweight or falling into obesity, nutrition and dietary modification are always part of disease management. It is not specifically established that BMI puts patients with GCA at risk for CVE. However, studies have identified an inverse relationship: a 0.1 kg/m^2^ decrease in BMI tends to increase the 8% higher risk of having a CVE in GCA [22]. A recent study that conducted a trial on GCA patients reported that a change in BMI was not associated (*p* = 0.27) with the disease relapsing among already‐diagnosed GCA patients [23]. Having an immediate effect on the state of the disease, BMI needs to be studied in more detail, highlighting every aspect of BMI change. A longitudinal cohort is also needed, as the observational studies lack causation. A study found that patients with CIEs in GCA patients maintained an average BMI of 22.5 kg/m^2^, while those without CIEs had 24.9 kg/m^2^. Further research is needed to determine safe BMI ranges for CIE prevention [6]. It can be hypothesized that helping GCA patients to raise their BMI may protect the patient from GCA‐related CIEs.

### 2.3 Anemia

A retrospective study published in 2009 on 287 GCA cases with biopsy evidence provided fascinating new information on the connection between anemia and CIE. Surprisingly, only one patient out of seven with vertebrobasilar stroke showed anemia (hemoglobin < 12 g/dL), compared with 56.1% of people without stroke. A significant reduction in the risk of stroke was observed in patients with GCA, confirmed by biopsy who were anemic at the time of diagnosis (OR, 0.11; 95% CI, 0.04–0.32; *p* < 0.001) and in women (OR, 0.10; 95% CI, 0.04–0.26; *p* < 0.001). Additionally, individuals with anemia (hemoglobin < 12 g/dL) at the time of GCA diagnosis had a lower chance of vertebrobasilar strokes (OR, 0.13; 95% CI, 0.04–0.47; *p* = 0.002). This study highlights the intricate interactions between anemia and stroke risk in GCA by focusing on biopsy‐proven cases across a 27‐year period. These results incline toward the fact that anemia may offer some protection against cerebrovascular problems in GCA patients [9].

In the 2021 pilot study, anemia (hemoglobin levels < 13 g/dL in men and < 12 g/dL in women) was 100% prevalent in patients with GCA and vertebrobasilar stroke. This result was consistent with the study, which discovered that anemia was a prevalent trait among stroke victims who had GCA. Anemia was significantly associated with GCA‐related stroke in the study population (10.4 against 14.6 mg/dL, *p* = 0.003). The literature review also indicated that female sex was protective against stroke in the setting of anemia in both the carotid and vertebrobasilar areas, although precise prevalence figures were not provided [14].

A retrospective multicenter cohort study comparing patients with GCA‐related stroke to a control group of GCA patients without stroke revealed anemia to be a significant contributing factor. The former were older [78 years (60–91) vs. 74 years (50–94), *p* = 0.03], had lower rates of anemia [22/37 (59%) against 137/167 (79%), *p* < 0.001, and had greater symptoms of ocular ischemia [25 (63%) vs. 50 (25%), *p* < 0.001). In addition, the ones with stroke had a decreased quantity of inflammatory markers; they also had an elevated hemoglobin quantity. These findings demonstrate the complex relationships between age, clinical symptoms, hematologic parameters, and inflammatory status that exist in the context of a GCA‐related stroke [11]. It can be hypothesized that lowering hemoglobin levels may protect against GCA‐related CIE.

### 2.4 Thromboembolism

The pathogenesis behind the thromboembolism events in GCA patients is not fully understood, but it is believed to be linked with systemic inflammation since GCA is a chronic inflammatory disease [24], affecting large to medium‐sized arteries due to immune system dysregulation, the pathophysiology affects the vascular system, leading to vascular injury [25]. The systemic immune response may modulate a thrombotic response due to dysregulation of anticoagulants suppressing fibrinolysis [26]. Inflammatory vascular conditions also damage the vascular endothelium lining, which contributes to thromboembolism. Venous thromboembolism incidence in GCA patients was found to be 13.3/1000/year in a cohort, population‐based study [26]. Studies have shown that patients who were recently diagnosed with GCA were more likely to experience venous thromboembolism in the first 3 months of the diagnosis [27]. The limitations reported for these studies relating to GCA and thromboembolism include that the data reported was limited to inpatient retrospective records, which lacked detailed clinical information, limiting the generalization of the population and leading to biases in result interpretation [28].

### 2.5 Platelets

The relationship between the platelets and cerebral ischemic events in GCA patients is well understood, but there is evidence found indicating thrombocytosis and the presence of higher platelets as a potential risk factor leading to CVE in GCA patients (HR 3.09, *p* = 0.0009) [29]. However, a study has reported that a higher incidence of CIE in GCA patients was not associated with platelet size and count (*p* = 0.56; OR 1.13 (0.75–1.69)) [30]. These studies had heterogeneity in the patient population, limited prospective studies, a small sample size, different diagnostic criteria, and a lack of standardized platelet measurement. A study has shown that platelet levels above 300 × 109/L are often used as diagnostic criteria of GCA; higher platelet levels lead to a procoagulant state leading to CIE [31].

### 2.6 Antiplatelet Therapy

The relationship between thromboembolic events in GCA patients highlights the importance of antiplatelet or anticoagulant therapy to be studied, as these are among the primary management strategies for thromboembolic events [32]. A study reported that 16.2% of diagnosed GCA patients compliant of antiplatelets had CIE, whereas in 48% of their study population, those who were not compliant with it had CIE, indicating antiplatelet medication as a potential factor (*p* = 0.0005) in decreasing the incidence of CIE in GCA [33]. Antiplatelet therapy lowers the risk of having CIEs, and a study showed an odds ratio of 0.318 without significantly increasing bleeding risk [34].

### 2.7 Aspirin

The role of aspirin in GCA patients has been the subject of research, focusing on its potential benefits as an adjunctive treatment. Being an anti‐inflammatory and antiplatelet agent, it has shown a decreased risk of cerebral ischemic complications in GCA patients. A study showed that at diagnosis, 8% of the GCA patients taking low‐dose aspirin, typically 100 mg/day, had CIE events, whereas among those who were not taking aspirin, 29% had CIE complications. Further, a study revealed that the therapeutic effects of aspirin were significantly connected (*p* = 0.028) with the CIE complications of GCA [28]. However, some studies failed to establish a statistical association between these variables due to a lack of homogeneity, a scarcity of randomized control trials (RCT) meeting the specific criteria, a retrospective design, and various confounding variables [28].

### 2.8 Inflammatory Markers

According to ACR recommendations [4], certain markers—such as CRP ≥ 10 mg/L or ESR ≥ 50 mm/h—are considered clinically significant. However, several intriguing findings have been revealed by the research that is covered here.

A significant finding was observed in a prospective research by Alojzija Hočevar [13] and colleagues in 2020, which included 295 patients with GCA. A modestly decreased threat of serious cardiovascular ischemic complications (sCIC) was linked to higher levels of CRP [Odds Ratio (OR) 0.99, 95% Confidence Interval (CI): 0.99–1.00, *p* = 0.011]. However, it is crucial to acknowledge the particular limitations that are inherent in this study. Notably, the study was only conducted in one place, which could add bias and restrict how broadly the findings can be applied. Additionally, relying on histology or imaging modalities improved the diagnosis's robustness, enhancing the study's findings' validity and accuracy. This study contributes to the body of literature by examining the relationship between elevated CRP levels in GCA patients and a lower risk of sCIC. Nonetheless, a cautious interpretation is required due to the single‐center design's constraints. The advantages of the study, including its broad cohort size and stringent diagnostic criteria, contribute to a deeper comprehension of the relationship between cardiovascular outcomes and CRP in GCA patients.

To try to assess the potential protective function of pro‐inflammatory cytokines in avoiding ischemia events in GCA, Hernandez and colleagues [35] carried out prospective cohort research. In GCA patients with a biopsy‐verified diagnosis, the study comprised 66 individuals for tissue expression analysis and 80 people for circulating levels measurement of interleukin (IL)‐1β, tumor necrosis factor‐α (TNF‐α), and IL‐6. The levels of circulatory cytokines were measured using an enzyme‐linked immunoassay, and the expression of tissue cytokines was found by immunohistochemistry and quantitative real‐time polymerase chain reaction. Comparing GCA patients with disease‐related ischemia episodes to those without ischemic sequelae, the findings showed lower circulating levels of IL‐6, lower IL‐6 immunohistochemistry expression scores, and lower IL‐6 mRNA levels. Remarkably, no appreciable variations were found in TNF‐α or IL‐1β. Subsequent investigations looked into how IL‐6 directly affected the components of the vessel wall. In both ex vivo (aortic ring) and in vivo (chick chorioallantoic membrane) testing, the study discovered that IL‐6 produces full angiogenic activity, increases endothelial cell proliferation, and promotes differentiation into capillary‐like structures [35].

In summary, GCA patients with ischemic outcomes exhibited lower tissue expression and circulating levels of IL‐6 in comparison to those without ischemia episodes. According to the study, IL‐6 directly affects arterial wall components and may have protective effects by triggering angiogenesis‐related functional programs. In patients with GCA, this possible compensatory mechanism through IL‐6‐induced angiogenesis may help to lessen ischemia episodes [35].

Hubert de Boysson oversaw a retrospective multicenter cohort study [11] involving individuals with a diagnosis of GCA. When GCA patients without stroke were compared with those who had the illness, the latter group had a lower prevalence of anemia (59% vs. 79%, *p* = 0.03) and a lower count of biological inflammatory markers, such as CRP (61 mg/L, interquartile range 28–185, vs. 99 mg/L, interquartile range 6–400, *p* = 0.04). These results are comparable with those of Cid et al., who likewise observed lower ESR and higher hemoglobin levels in those who experienced bouts of cerebral ischemia [11]. These findings are consistent with earlier research that discovered lower levels of IL‐6, a cytokine essential to the pathophysiology of GCA, in situations linked to ischemia symptoms related to GCA. Moreover, endothelial and smooth muscle cells may be significantly impacted by the local production of pro‐inflammatory cytokines in inflamed arteries, such as IL‐1β, IL‐6, and TNF‐α. These actions may have an effect on vascular tone modulation, myointimal cell proliferation, procoagulant activity, neovascularization, and matrix deposition. Thus, endothelial cells and the control of vascular responses may be impacted by the regulation of inflammation. It is important to recognize some of the study's shortcomings, though. First of all, it excluded other cerebrovascular episodes that are suggestive of brain vessel involvement, such as TIA or multi‐infarct dementia. Furthermore, it was difficult to get complete data for every patient because of the retrospective design, especially for the control group.

Miguel Gonzalez‐Gay conducted a retrospective review of case records involving GCA patients whose GCA was verified by biopsy. The goal of the research revolved around finding out the potential connection between clinical features, GCA test indicators, and the risk of stroke. ESR levels in stroke patients (81.6 ± 20.0 mm/1st h vs. 93.8 ± 22.7 mm/1st h, respectively) were lower than in nonstroke patients at the point of confirmation of the disease; however, the difference was considerably impactful (*p* = 0.10). The study's findings demonstrated that there was a noticeably greater risk of permanent blindness among GCA patients who experienced strokes. Importantly, these cerebrovascular strokes occurred shortly after the development of ocular symptoms [9]. Consequently, it was discovered that having irreversible blindness was a robust indicator of strokes, establishing a direct link between eye issues and subsequent CVE. It's interesting to note that anemia was found to have an inverse relationship with protection against the emergence of these neurological issues at the time of confirmation of the disease. In the context of GCA, this suggests that anemia may have little impact on lowering the risk of strokes.

A multicenter retrospective study involving 129 patients diagnosed with GCA was conducted under the direction of Aaron Pariente [12]. The investigation did not identify any major shifts in CRP levels, the frequency of positive temporal biopsies, or clinical features comparing GCA patients with and without stroke at the time of GCA confirmation [12].

Having said that, there are some restrictions to be mindful of. First, biases might have been created by the study's retrospective design. Furthermore, the restricted generalizability of the data may have been caused by the exceedingly small sample size of stroke patients. It's critical to remember that the risk variables linked to the frequency of stroke in GCA patients can only be conclusively determined by prospective research. To fully comprehend the prevalence and risk factors of strokes in people with GCA, more prospective research is needed.

### 2.9 Tocilizumab

As an IL‐6 receptor antagonist, tocilizumab [TCZ], a humanized monoclonal antibody, inhibits the inflammatory signaling pathway, limiting vascular endothelial damage, preventing inflammation from progressing, and indirectly preventing the activation of the clotting cascade, all of which reduce the risk of thromboembolic events. It is hence used in the long‐term treatment of GCA, as it also has a steroid‐sparing effect [36]. Studies have indicated a substantial (*p* = 0.001) correlation between the usage of TCZs and the annualized flare rate of GCA. The flare rate was 1.4 [95% CI 1.0–2.1] events/year before using TCZ, and the incidence of new vision symptoms was likewise reduced to 0.6 [95% CI 0.3–1.0] events/year following TCZ treatment [37]. One‐third of patients experienced mild to moderate adverse effects from tocilizumab, which can be used to lower glucocorticoid (GS) exposure in the population, according to studies evaluating the safety and efficacy of the medication in GCA patients over the age of 80 [38]. To establish a strong association and its adverse effect, we require more studies at a larger scale, studying for longer durations on different age groups, focusing on clinical remission and relapse, and having standardized diagnostic criteria for GCA and its complications.

### 2.10 Glucocorticoid

Glucocorticoid has been a cornerstone treatment in GCA patients as it reduces inflammation, decreasing the inflammatory response and endothelial injury. A study reported that 80% of glucocorticoid therapy in patients leads to toxicity [39]. The established pharmacodynamics of glucocorticoid, as they exacerbate hypertension and dyslipidemia, act as confounding variables in the development of CVE in GCA‐diagnosed patients [40]. A cohort conducted on GCA patients reported that 32% of the patients, of whom 19% stopped their glucocorticoid at 1 year of treatment, had a rapid tapering regimen. Most GCA relapses were minor with glucocorticoid, where 40% of their patients were found to have adverse effects [41]. A study conducted to reveal the clinical outcomes of long‐term use of glucocorticoids, particularly prednisolone, showed that there were significant chances (*p* = 0.05) of an increasing trend to develop diabetes mellitus and osteoporosis [42]. It will take more extensive, prospective follow‐up studies to prove a cause‐and‐effect link between glucocorticoid therapy and clinical outcomes.

### 2.11 Other Risk Factors

Apart from the above‐mentioned risk variables, there are additional considerations that could lead to GCA‐induced CIA, including age, male gender, and social deprivation [43], as well as conventional cardiovascular risk factors like smoking, diabetes, and atherosclerosis that may result in GCA‐induced CIA. Twenty‐four of the 285 GCA‐diagnosed CVE patients in a Spanish observational study conducted in 2023 were male. They discovered that male gender (OR = 1.35, 95% CI 1.06–1.72) and hypertension (OR = 1.44, 95% CI 1.11–1.90) were linked to an increased likelihood of developing GCA‐induced CIE in their multivariate analysis [44]. GCA often manifests after the age of 50 and is most commonly observed in people who are over 70 [17]. Therefore, GCA‐related CIE typically affects older people. A meta‐analysis indicates that, in comparison to non‐GCA patients, the risk ratio of GCA‐related CIE is 1.40 [95% CI: 1.27–1.56].

Another study in 2009 found smoking history to be significantly associated with patients who developed strokes due to GCA when compared with the ones with no smoking history (50.0% vs. 14.9%, respectively; *p* = 0.02). In fact, this study found smoking history to be the best predictor of strokes involving the vertebrobasilar arterial system [9].

Traditional cardiovascular risk factors still have debates about their relevance; thus, further study is necessary to determine their effects. For example, hyperglycemia induces increased PD‐L1 expression in macrophages, which suppresses CD4 + T cell proliferation, reducing the inflammatory response in GCA patients. Macrophages in atherosclerotic plaques and monocyte‐derived macrophages in coronary artery disease patients also increase PD‐L1 expression [43]. In GCA, granulomatous inflammation forms in the artery alongside intimal hyperplasia, causing stenosis and thrombosis, which precipitate into ischemia, leading to stroke [18]. Due to the reduced CD4 + T cell proliferation, the chances of vasculitis‐induced stenosis are reduced [17] and immune cells causing luminal compromise via inflammation are reduced [18]. However, these traditional cardiovascular risk factors can cause thrombosis and embolism, which may lead to stroke (Table 2).

**Table 2:** Summary of risk factors associated with giant cell arteritis (GCA) and cerebrovascular ischemic events (CIE).

Risk factors	Description
Hypertension	Significant correlation between hypertension and stroke in GCA patients; every stroke patient with GCA had hypertension; men accounted for 75% of GCA‐related stroke cases; vertebrobasilar region primary site of strokes; incidence rates varied by gender and age.
BMI	Variations in BMI linked to elevated risk of cerebrovascular events in GCA patients; BMI change not linked to disease relapse in diagnosed GCA patients.
Anemia	Anemia at GCA diagnosis linked with lesser chance of stroke; common in patients with vertebrobasilar stroke and GCA; significantly linked to GCA‐related stroke; hemoglobin levels lower in GCA related stroke patients compared with those without stroke; studies highlight importance of close monitoring and individualized treatment plans for anemic GCA patients.
Other risk factors	Additional risk factors for GCA‐induced cerebrovascular ischemic events (CIE) include age, male gender, and social deprivation; smoking history significantly associated with strokes due to GCA; hyperglycemia, atherosclerotic plaques and coronary artery disease(CAD) may impact inflammatory response in GCA patients; traditional cardiovascular risk factors can lead to thrombosis and embolism, potentially causing stroke in GCA patients; further research needed to establish impact of traditional cardiovascular risk factors on GCA‐related CIE.

## 3 Recommendations

In individuals with GCA, cerebrovascular episodes are one of the causes listed as the primary causes of death. In the days or weeks that followed, 28% of patients with GCA‐related strokes passed away, according to a multicenter retrospective analysis [11]. In another study, 2 weeks after GCA diagnosis, the incidence of ischemic events was found to be 74% for transient (jaw claudication, amaurosis fugax, TIA) and irreversible events, and 34% for severe irreversible events (anterior ischemic optic neuropathy and stroke) [30].

This information highlights the importance of physicians being cautious for a CIE to occur in a newly diagnosed GCA patient. Physicians should closely monitor newly diagnosed GCA patients for CIE and develop management plans to minimize the risk of GCA‐related CIEs in the first few days of diagnosis. GCA patients may be treated aggressively for GCA‐related CIE when they present with recent ophthalmic ischemic symptoms, particularly in newly diagnosed GCA patients. Vasculitis is more probable as the cause of strokes in newly diagnosed GCA patients; hence, this should be considered while developing a management plan [11]. Since GCA patients are more likely to experience venous thromboembolism in the first 3 months after diagnosis [27], anticoagulant therapy and low‐dose aspirin may be given to prevent CIE. Care must be taken to prevent adverse effects of anticoagulant therapy. Aggressive treatment may be stopped after GCA goes into remission, during which drugs such as tocilizumab may be given to not only maintain remission but also reduce the risk of GCA‐related CIE.

GCA must be kept in mind when managing patients over 50, especially those who come with visual symptoms like blurred vision, amaurosis fugax, diplopia, and jaw claudication. Symptoms including a history of vision loss and risk factors like male gender, high hemoglobin levels, hypertension, and smoking are essential to be considered when evaluating the risk of stroke in GCA patients. To receive prompt medical attention, patients and GCA attendants need to be aware of signs and symptoms of stroke, such as the FAST acronym, which identifies facial droop, arm drift, and slurred speech as common signs and symptoms of stroke, and the symptoms mentioned above, which are unique clinical manifestations of GCA‐related stroke, so that they can immediately seek medical attention as they are at higher risk of stroke. Another aspect to keep in mind when dealing with patients over 50 who appeared with stroke is that they may present with GCA‐related stroke without prior GCA diagnosis, due to which they may not meet the ACR criteria as stroke was excluded from the criteria [18]. Physicians should consider a history of headaches, arthritis, and elevated levels of markers of inflammation such as CRP and ESR to be suggestive of a GCA‐related stroke. If the halo sign in duplex ultrasonography of cervical vessels is found, it may also indicate a GCA‐related stroke [15]. When treating GCA patients with glucocorticoids, physicians must consider that long‐term glucocorticoid therapy raises the chances of stroke as an adverse effect. Tocilizumab may be considered an alternative therapy to glucocorticoid therapy. Antiplatelet therapy such as aspirin and warfarin may be given to patients to prevent ischemic stroke, although more research is needed to study their protective effects.

When stroke patients are present in an emergency, especially with a history of GCA diagnosis, imaging techniques should be used to assess the degree of damage caused by the ischemic event. The techniques include, for instance, magnetic resonance imaging, computed tomography, ultrasonography, and Doppler's scan. Physicians should keep in mind that GCA‐related strokes can present in the vertebral and basilar arteries and in the carotid artery. Even though intracranial artery involvement is thought to be uncommon, current research has revealed that it is more widespread than previously thought [45, 46], physicians should therefore take this into account when prescribing imaging procedures. Strokes associated with GCA have been found to be strongly predicted by ischemic events in the ocular artery. The greatest predictor of GCA‐related CIE among the clinical signs of ischemic ophthalmic events is irreversible blindness [7].

## 4 Future Perspectives

Further research can be done to establish safe and recommended ranges for risk factors such as BMI, platelets, hemoglobin, and inflammatory markers in GCA patients for physicians to target to prevent CIE. These risk factors alongside age, smoking, gender and hypertension can be used to quantify how likely a GCA patient is to experience a GCA‐related CIE in the near future. Research can also be done to develop optimum doses that minimize the risk of adverse effects such as hemorrhage from anticoagulation therapy while also preventing CIE in GCA patients.

## 5 Conclusion

To sum up, this narrative study offers insightful information about the intricate nature of CIEs associated with GCA/cerebrovascular ischemia. By exploring common risk variables such as age, gender, hypertension, and smoking and examining protective factors such as anemia and specific inflammatory markers, this study contributes to a more thorough knowledge of the dynamics driving GCA‐related CIE. The discovery that the signs and symptoms of ophthalmic artery ischemia are a reliable indicator enhances our capacity to evaluate and treat GCA patients' risks.

In addition, the study emphasizes the importance of pharmaceutical therapies by stressing the effectiveness of antiplatelet medication, glucocorticoids, tocilizumab, and aspirin in the treatment and prevention of CIE. To establish strong connections, rigorous research is required, as highlighted by the requirement for comprehensive and suitable study designs for risk variables.

Ultimately, the knowledge distilled from this narrative review not only enriches our comprehension of GCA and its complications but also equips healthcare professionals with actionable insights for risk assessment and preventive strategies. The results highlight how crucial proactive care is for improving patient outcomes and lessening the effects of CIE in the setting of GCA.

## Author Contributions

**Muhammad Osama Siddiqui:** conceptualization, writing and reviewing. **Mohammad Ali Syed:** conceptualization, writing–review and editing, drafting, revisions, supervision. **Ayaan Ahmed Qureshi:** conceptualization, writing–review and editing, drafting, revisions, supervision. **Mustafa Hussain Imam:** writing–review and editing, drafting, revisions, supervision. **Jatin Motwani:** writing and reviewing. **Verkha Kumari:** writing and reviewing. **Arooba Siddiqui:** writing and reviewing. **Noor Ul Ain:** writing–review and editing. **Mohammed Hammad Jaber:** reviewing.

## Ethics Statement

The authors have nothing to report.

## Conflicts of Interest

The authors declare no conflicts of interest.

## Figures and Tables

**Figure A1:**
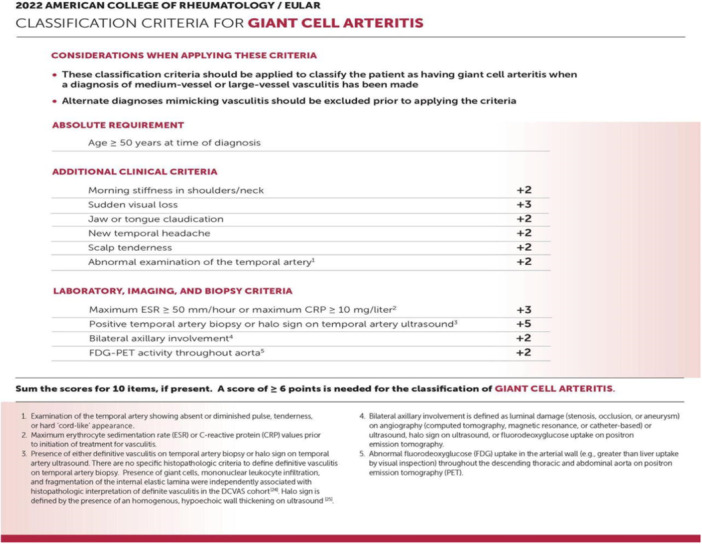
2022 ACR/EULAR criteria for diagnosing GCA [4].

## Data Availability

The authors have nothing to report.

## Appendix A

See Figure A1.
